# Effects of the Cytoplasm and Mitochondrial Specific Hydroxyl Radical Scavengers TA293 and mitoTA293 in Bleomycin-Induced Pulmonary Fibrosis Model Mice

**DOI:** 10.3390/antiox10091398

**Published:** 2021-08-31

**Authors:** Takahiro Sakai, Hidetsugu Takagaki, Noriyuki Yamagiwa, Michio Ui, Shinichi Hatta, Jun Imai

**Affiliations:** Laboratory of Physiological Chemistry, Faculty of Pharmacy, Takasaki University of Health and Welfare, 60 Nakaorui-machi, Takasaki 370-0033, Japan; hide.takagaki@gmail.com (H.T.); yamagiwa@takasaki-u.ac.jp (N.Y.); ui-mc@igakuken.or.jp (M.U.); hattaeb1406@gmail.com (S.H.); jimai@takasaki-u.ac.jp (J.I.)

**Keywords:** hydroxyl radical, cytoplasmic hydroxyl radical, mitochondrial hydroxyl radical, cellular senescence, pulmonary fibrosis, inflammation

## Abstract

Lung fibrosis is the primary pathology in idiopathic pulmonary fibrosis and is considered to result from an increase in reactive oxygen species (ROS) levels in alveolar epithelial cells. However, the exact mechanism underlying lung fibrosis remains unclear and there is no effective therapy. The hydroxyl radical (^•^OH) has the strongest oxidizing potential among ROS. Recently, ^•^OH localized to the cytoplasm (cyto ^•^OH) was reported to induce cellular senescence, while mitochondria-localized ^•^OH (mt ^•^OH) was reported to induce apoptosis. We developed the cyto ^•^OH- and mt ^•^OH-scavenging antioxidants TA293 and mitoTA293 to evaluate the effects of cyto ^•^OH and mt ^•^OH in a bleomycin (BLM)-induced pulmonary fibrosis model. Treatment of BLM-induced pulmonary fibrosis mice with TA293 suppressed the induction of cellular senescence and fibrosis, as well as inflammation in the lung, but mitoTA293 exacerbated these. Furthermore, in BLM-stimulated primary alveolar epithelial cells, TA293 suppressed the activation of the p-ATM^ser1981^/p-p53^ser15^/p21, p-HRI/p-eIF2^ser51^/ATF4/p16, NLRP3 inflammasome/caspase-1/IL-1β/IL1R/p-p38 MAPK/p16, and p21 pathways and the induction of cellular senescence. However, mitoTA293 suppressed the induction of mitophagy, enhanced the activation of the NLRP3 inflammasome/caspase-1/IL1β/IL1R/p-p38 MAPK/p16 and p21 pathways, and exacerbated cellular senescence, inflammation, and fibrosis. Our findings may help develop new strategies to treat idiopathic pulmonary fibrosis.

## 1. Introduction

Idiopathic pulmonary fibrosis (IPF) is characterized by diffusive and rapidly developing fibrosis, resulting in impaired gas exchange, restricted ventilation problems, and eventual respiratory failure [1]. Therefore, the mortality rate associated with IPF is high [2,3]. In addition, the prevalence of IPF is estimated to be 14–43 per 100,000 worldwide [4,5]. Since fibrosis is irreversible, effective treatment for this disorder needs to be administered early before major lung destruction and fibrosis occur. Oxidative stress has been speculated to play a central role in the onset and exacerbation of IPF [6]. Reactive oxygen species (ROS) cause oxidative stress and inflammation and damage tissues [7]. Inflammation has been suggested to cause fibrosis via fibroblast proliferation and collagen deposition. However, in IPF, the effects of oxidative stress and inflammation on the induction and exacerbation of fibrosis remain unclear.

The bleomycin (BLM)-induced pulmonary fibrosis model is an experimental model of human IPF involving ROS [8]. BLM-induced ROS production is based on intracellular iron binding. The BLM–iron complex reduces molecular oxygen to a superoxide radical (O_2_^•−^) and hydroxyl radical (^•^OH). It has been suggested that BLM forms a complex with Fe (II) or Fe (III) and secondarily produces ^•^OH, which in turn damages lung tissue [9]. In the lungs of the BLM-induced pulmonary fibrosis model, generation of ROS results in lipid peroxidation, DNA injury, alterations in prostaglandin synthesis and degradation, and an increase in collagen synthesis [10,11]. ROS-induced oxidative damage in tissues induces fibrosis by acute inflammation, fibroblast infiltration, and collagen production [12]. Among the intracellular ROS, O_2_^•−^, hydrogen peroxide (H_2_O_2_), and ^•^OH, are produced in sequence [13]. To date, in BLM-induced pulmonary fibrosis models, ROS-induced oxidative damage has been inhibited, at least in part, by the addition of various antioxidants [14,15,16]. However, since these antioxidants non-specifically scavenge all ROS, they suppress the immune response and redox signal regulation by O_2_^•−^ and H_2_O_2_, and it is highly possible that they inhibit the maintenance of homeostasis. Among the ROS, ^•^OH, with the strongest oxidative potential, plays a central role in oxidative damage to cells [17,18,19]. Therefore, ^•^OH is presumed to be the source of oxidative stress in the BLM-induced pulmonary fibrosis model. However, the effect of ^•^OH on lung fibrosis in this model is unknown. Furthermore, ^•^OH does not diffuse inside cells, unlike other ROS, owing to its high reactivity [17,19]. Consequently, it is inferred that the intracellular compartment that produces ^•^OH is specifically oxidatively damaged by ^•^OH itself. Recent reports have suggested that differences in these intracellular compartments can cause different pathophysiological effects [17,20,21,22]. In the BLM-induced pulmonary fibrosis model, differences in the intracellular compartments oxidatively damaged by OH are expected to have different effects on the induction of fibrosis, but these effects have not been clarified.

Based on these findings, we developed the cyto ^•^OH- and mt ^•^OH-targeted antioxidants TA293 and mitoTA293 to clarify the effects of cyto ^•^OH and mt ^•^OH in a BLM-induced pulmonary fibrosis model in vivo and in vitro. Here, we report a novel mechanism by which cyto ^•^OH and mt ^•^OH respectively induce or suppress cellular senescence, inflammation, and fibrosis in a BLM-induced model.

## 2. Materials and Methods

### 2.1. Reagents

TA293, a cyto ^•^OH-targeted antioxidant was designed based on the structure of ascorbic acid in our laboratory and was developed by DIC Corporation (Chiba, Japan) [17,23] (Supplementary Figure S1). MitoTA293, in which the mitochondrial localization signal triphenylphosphonium is added to TA293, was synthesized by Takasaki University of Health and Welfare (Gunma Prefecture) [17,23] (Supplementary Figure S1). BLM was procured from Nippon Kayaku (Tokyo, Japan). Antimycin A was purchased from Sigma-Aldrich (St. Louis, MO, USA).

### 2.2. Mice

We purchased C57BL/6J mice from CLEA Japan, Inc. (Tokyo, Japan) and Keap1-based oxidative stress detector 48-transgenic (OKD48-Tg) and IL-1β-based dual-operating luciferase transgenic (IDOL-Tg) albino C57BL/6J mice from TransGenic Inc. (Kobe, Japan) [24,25]. Only 12-week-old male mice were used in the experiment. However, the sex of mice was not expected to affect the results. Mice were intratracheally treated with BLM (20 mg/kg) using a microsprayer (Penn-Century, Philadelphia, PA, USA). TA293 (10 mg/kg) or mitoTA293 (10 mg/kg) was sprayed intratracheally using a microsprayer once a day from day 0 to day 7 (*n* = 5 in each group).

The animal studies in this study were strictly in compliance with the Guide for the Care and Use of Laboratory Animals by the US National Institutes of Health (NIH Publications No. 8023, revised 1978), and were approved by the Animal Experiment Ethics Committee of Takasaki University of Health and Welfare, Japan.

### 2.3. Cell Culture and Drug Treatment

Primary pulmonary alveolar epithelial cells (PAECs) isolated from the lungs of C57BL/6J mice were obtained from Cell Biologics (Chicago, IL, USA). The PAECs were cultured in vitro in complete medium comprising base medium and supplements (Cell Biologics) in 5% CO_2_ at 37 °C. The PAECs were passaged 4–10 times before use in experiments. To test the cellular response to drugs, PAECs were treated with either 10 μg/mL BLM or 2 μg/mL antimycin A and either 100 μM TA293 or 100 μM mitoTA293 for 72 h.

### 2.4. Measurement of Senescence-Associated β-Galactosidase Activity

Senescent cells in lungs collected from mice 8 days after BLM administration and senescent PAECs 3 days after BLM stimulation were assessed by senescence-associated β-galactosidase (SA-β-gal) staining using the Senescence Detection Kit (BioVision, Mountain View, CA, USA) following the manufacturer’s instructions. Briefly, frozen tissue sections and PAECs on glass coverslips were fixed in 2% formaldehyde and incubated with staining solution for 16 h at 37 °C. Subsequently, the slides were washed with phosphate-buffered saline (PBS) and mounted using Permount (Fisher Scientific, Bridgewater, NJ, USA). Finally, positively stained cells were counted under an NIS-Elements microscope (Nikon Instruments, Tokyo, Japan) at 20× magnification in five random fields for each experimental condition, and SA-β-gal positive cells were quantified.

### 2.5. RNA Isolation and Quantitative RT-PCR

Total RNA was extracted from the lung tissue of mice and PAECs using the ISOGEN Regent (Takara Bio, Kyoto, Japan) according to the manufacturer’s protocol. The reverse transcription of the extracted RNA (1 μg) was performed using the RNA LA PCR Kit (Takara Bio), and mRNA expression was analyzed by qRT-PCR using the Thunderbird SYBR qPCR Mix (Toyobo, Osaka, Japan) and previously described primers [17]. Five replicates were used in each experiment.

### 2.6. Western Blotting

Lung tissue and cells were homogenized on ice in RIPA buffer containing protease and phosphatase inhibitors (Nacalai Tesque Inc., Kyoto, Japan) and the homogenate was centrifuged at 11,000× *g* for 30 min at 4 °C. The protein concentration in the supernatant was measured by Bradford assay (Bio-Rad, Hercules, CA, USA). Western blotting was performed following standard procedures, using primary antibodies against p16INK4a (1:1000, BS1265, Bioworld Technology, St. Louis Park, MN, USA), p21Waf1/Cip1 (1:1000, sc-6246, Santa Cruz Biotechnology, Dallas, TX, USA), α-SMA (1:1000, #14968, Cell Signaling Technology, Beverly, MA, USA), COL1A1 (1:1000, #8433, Cell Signaling Technology, Beverly, MA, USA), ATF4 (1:1000, #11815, Cell Signaling Technology, Beverly, MA, USA), p-p53^ser15^ (1:1000, #9284, Cell Signaling Technology, Beverly, MA, USA), p-eIF2^ser51^ (1:1000, #9721, Cell Signaling Technology, Beverly, MA, USA), NLRP3 (1:1000, #15101, Cell Signaling Technology, Beverly, MA, USA), p-p38MAPK (1:1000, #9211, Cell Signaling Technology, Beverly, MA, USA), β-actin (1:1000, #4967, Cell Signaling Technology, Beverly, MA, USA), p-ATM^ser1981^ (1:1000, 200-301-500, Rockland Immunochemicals Inc., Limerick, PA, USA), PINK1 (1:1000, 23274-1-AP, Proteintech, Chicago, IL, USA), Parkin (1:1000, 14060-1-AP, Proteintech, Chicago, IL, USA), and HRI (1:1000, 07-728, Merck Millipore, Bedford, MA, USA). After incubation with anti-rabbit horseradish peroxidase (HRP)-conjugated secondary antibody (1:2000, #7074, Cell Signaling Technology, Beverly, MA, USA) and anti-mouse HRP-conjugated secondary antibody (1:2000, #7076, Cell Signaling Technology, Beverly, MA, USA), signals were detected using ECL Plus Western Blotting Detection Reagents (GE Healthcare Life Sciences, Piscataway, NJ, USA).

### 2.7. In Vivo Imaging Analysis

OKD48- and IDOL-Tg mice were injected intraperitoneally with D-luciferin (OZ Biosciences, San Diego, CA, USA) reconstituted in PBS (150 mg/kg body weight), 3 or 8 days after BLM administration with or without TA293, mitoTA293, or vehicle (saline) (*n* = 5/group). Oxidative stress and inflammation in the lungs of these mice were detected using the IVIS Lumina II in vivo imaging system according to standard protocols (Caliper Life Sciences, Hopkinton, MA, USA).

### 2.8. Histological Analysis and Detection of Fibrosis

Histological analysis was performed on lungs fixed in 4% buffered formalin, cut into 3–5-μm thick sections, and stained with hematoxylin and eosin. Fibrosis was detected by Masson’s trichrome staining under an NIS-Elements microscope (Nikon Instruments) at 20× magnification. Fibrotic zones were quantitated using the NIS-Elements imaging software (Nikon Instruments).

### 2.9. siRNA Transfection

Cells were transfected with 10 nM of each siRNA using the Lipofectamine RNAiMAX transfection reagent (Life Technologies, Carlsbad, CA, USA) according to the manufacturer’s instructions. *Eif2ak1* (also called *Hri*), *Atm*, *Pink1*, *Parkin*, *Il1b*, *Il18*, *Nlrp3*, *Slc25a11*, and Control siRNA were purchased from Santa Cruz Biotechnology (Dallas, TX, USA). Knockdown efficiency was analyzed by qPCR-based gene expression analysis (Supplementary Figure S2).

### 2.10. Caspase-1 Activity Assay

The activity of intracellular caspase-1 was evaluated using the Caspase-Glo^®^ 1 inflammasome assay (Promega, Madison, WI, USA) according to the manufacturer’s instructions. This assay is a homogeneous and bioluminescent method to selectively measure the activity of caspase-1. Briefly, after removing half (50 µL) of the medium in the wells (5 × 10^4^ cells per well), 50 µL of the Caspase-Glo^®^ 1 reagent was added, and the solution was incubated at 22 °C for 1 h. Luminescence was measured using a luminometer (Berthold Technologies, Bad Wildbad, Germany).

### 2.11. Detection of Mitophagy

Mitophagy was detected using the Mitophagy Detection Kit (Dojindo Molecular Technologies, Kumamoto, Japan) according to the manufacturer’s protocol; cells were treated according to the manufacturer’s recommendations. Briefly, cells were washed twice with Dulbecco’s modified Eagle medium (DMEM) and incubated at 37 °C for 30 min with 100 nmol of Mtphagy Dye diluted in DMEM. After incubation, the cells were again washed twice with DMEM, followed by the addition of BLM and TA293 or mitoTA293. Subsequently, cells were trypsinized, and the fluorescence intensity of Mtphagy Dye was measured by flow cytometry at an excitation wavelength of 561 nm and emission wavelength of 570–700 nm.

### 2.12. Subcellular Fractionation 

The mitochondrial/cytosol fraction kit (BioVision Inc., Milpitas, CA, USA) was used to prepare the mitochondrial and cytosol fractions of cells. Briefly, cells washed with ice-cold PBS were resuspended in a Cytosol Extraction Buffer Mix containing dithiothreitol and a protease inhibitor, incubated on ice for 10 min, and then homogenized. The homogenate was centrifuged at 4 °C for 10 min (700× *g*), and the supernatant was separated and centrifuged at 1200× *g* at 4 °C for 30 min. The supernatant (cytosol fraction) was collected, and the pellet was resuspended in mitochondrial extraction buffer (mitochondrial fraction).

### 2.13. Measurement of Lipid Peroxide, H_2_O_2_, and ^•^OH in Mitochondrial and Cytosolic Fractions

Cells were treated with hydrogen peroxide target fluorescent probe Spy-LHP (Dojindo Laboratories, Kumamoto, Japan), BES-So-AM (Wako Pure Chemicals, Osaka, Japan), and hydroxyphenyl fluorescein (Sekisui Medical, Tokyo, Japan) at 37 °C for 30 min and washed with PBS. The supernatant was then removed. Mitochondrial and cytosolic fractions were prepared using a mitochondria/cytosol fractionation kit (BioVision Inc., Milpitas, CA, USA), and the fluorescence was detected using a microplate reader (Wallac 1420 ARVO MX Multilabel Counter; Perkin-Elmer, Waltham, MA, USA).

### 2.14. Measurement of 8-hydroxy-2′-deoxyguanosine 

Nuclear DNA (nDNA) and mitochondrial DNA (mtDNA) were extracted from the cytosolic and mitochondrial fractions using a DNA Extractor TIS kit (Wako Pure Chemicals). The obtained DNA was treated with nuclease P1 and alkaline phosphatase according to the manufacturer’s protocol. The level of 8-hydroxy-2′-deoxyguanosine (8-OHdG) in DNA was determined according to the manufacturer’s protocol by using an 8-OHdG ELISA kit (JaICA, Shizuoka, Japan) and measuring absorbance at 450 nm with a Sunrise microtiter plate reader (Tecan Japan, Kanagawa, Japan).

### 2.15. Quantitation of Cytosol and Mitochondrial Glutathione

Using a glutathione (GSH) quantitation kit (Dojindo Laboratories), cytosolic and mitochondrial GSH levels were determined according to the manufacturer’s instructions. Briefly, 20 μL of 5% salicylic acid was added to 80 μL of cytosol and mitochondrial fraction, and the mixture was centrifuged at 8000× *g* for 10 min at 4 °C. The GSH level in the supernatant was measured at 405 nm using a Sunrise Absorbance Microtiter Plate Reader (Tecan Japan).

### 2.16. Measurement of Mitochondrial Membrane Potential

Mitochondrial membrane potential was assessed using the JC-1 MitoMP Detection Kit (Dojindo Laboratories), according to the manufacturer’s protocol. Briefly, PAECs (1 × 10^6^ cells/well) were incubated with JC-1 working solution at 37 °C for 30 min. Subsequently, cells were washed twice with PBS and imaging buffer was added. Fluorescence was detected using a Wallac1420 ARVO MX multi-label counter (Perkin-Elmer) and the ratio of fluorescence measured at 435 and 590 nm to that measured at 485 and 535 nm was calculated.

### 2.17. Hydroxyproline Assay

The Sensitive Tissue Hydroxyproline assay kit (QuickZyme Biosciences, Leiden, The Netherlands) was used to measure the collagen content in the lungs of mice. Briefly, lung tissue was homogenized with 100 μL of distilled water per 10 mg tissue, acid hydrolyzed with 100 μL of 12 N HCl at 100 °C for 20 h, and centrifuged at 13,000× *g* for 10 min. Hydroxyproline was measured according to the manufacturer’s protocol.

### 2.18. Quantitation of Intracellular Heme

Intracellular heme content of PAECs was quantified using a heme assay kit (Sigma-Aldrich). Briefly, 200 μL of heme reagent was added to each 50 μL sample, incubated at about 22 °C for 5 min, and then the absorbance was measured at 400 nm.

### 2.19. Statistical Analysis

The standard error of the mean (SEM) was calculated based on five independent experiments. Statistical analysis was performed using one-way analysis of variance (ANOVA) using Excel Statistics (BellCurve, Tokyo, Japan) and IBM SPSS Statistics (Tokyo, Japan). Statistical significance was set at *p* < 0.01 and *p* < 0.05.

## 3. Results

### 3.1. TA293 Suppresses Induction of Oxidative Stress, Inflammation, and Fibrosis, but mitoTA293 Exacerbates These Conditions in BLM-Induced Pulmonary Fibrosis

**Figure 1:**
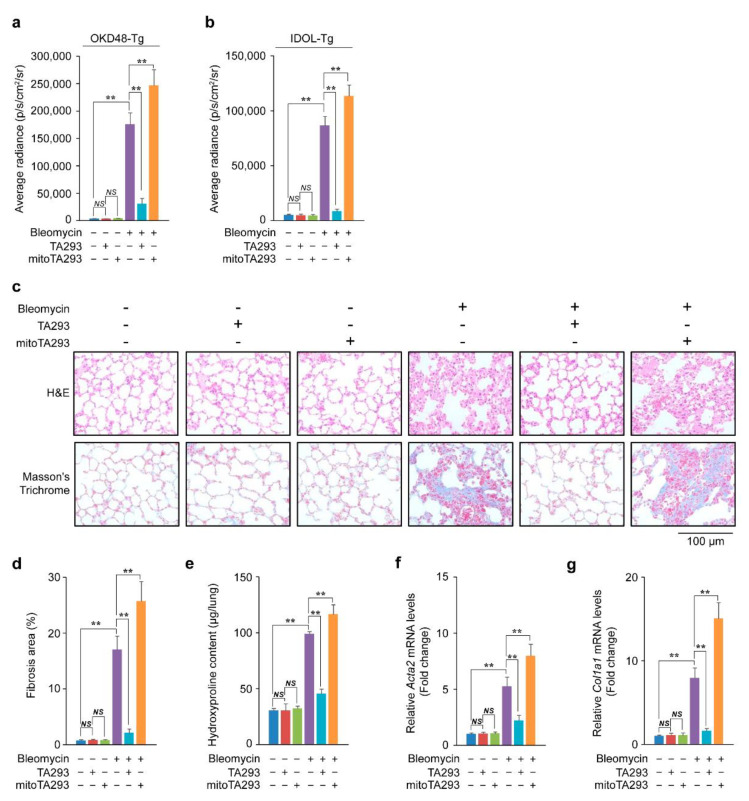
Effects of TA293 and mitoTA293 on oxidative stress, inflammation, and fibrosis in bleomycin (BLM)-induced pulmonary fibrosis model mice. Quantification of (**a**) oxidative stress and (**b**) inflammation in vivo. (**c**) Histopathology (hematoxylin and eosin (H&E) staining) and fibrosis (Masson’s trichrome staining) in mice 14 days after BLM administration. Pulmonary fibrogenesis was evaluated by semiquantitative analysis based on (**d**) Masson’s trichrome staining, (**e**) hydroxyproline content, and expression of (**f**) *Acta2* and (**g**) *Col1a1* mRNAs was determined using qRT-PCR. The data are presented as the mean ± SEM (*n* = 5 per group); ** *p* < 0.01; *NS*, not significant.

Based on the timeline shown in Supplementary Figure S3, TA293 and mitoTA293 were administered daily to the lungs of mice for seven days after BLM stimulation. In the lungs of OKD48-Tg mice three days after BLM stimulation, TA293 suppressed the increase in oxidative stress, but mitoTA293 exacerbated the oxidative stress (Figure 1a and Supplementary Figure S4a). In the lungs of the BLM-induced pulmonary fibrosis mouse model, the peaks of increased expression of inflammatory and fibrotic markers are observed around days 8 and 14, respectively [8]. Inflammation and fibrosis “switching” occurs approximately nine days after BLM administration [26]. In the present study, in the lungs of IDOL-Tg mice, eight days after BLM stimulation, TA293 suppressed the increase in inflammation, but mitoTA293 exacerbated inflammation (Figure 1b and Supplementary Figure S4b).

In the lung, 14 days after BLM administration, TA293 suppressed alveolar thickening, but mitoTA293 did not (Figure 1c). Furthermore, TA293 reduced the increase in Masson’s-stained areas and hydroxyproline content, while mitoTA293 further increased these (Figure 1c–e). In addition, TA293 suppressed the increase in expression of the fibrosis markers *Acta2* and *Col1a1α* mRNA, while mitoTA293 further increased their expression (Figure 1f,g).

Taken together, these results suggest that cyto ^•^OH induces oxidative stress and inflammation in the lungs of mice with BLM-induced pulmonary fibrosis, which leads to fibrosis. In contrast, mt ^•^OH attenuates oxidative stress and inflammation and suppresses the exacerbation of fibrosis.

### 3.2. In the Lungs of BLM-Induced Pulmonary Fibrosis Mice, TA293 Suppresses and mitoTA293 Exacerbates Cellular Senescence

**Figure 2:**
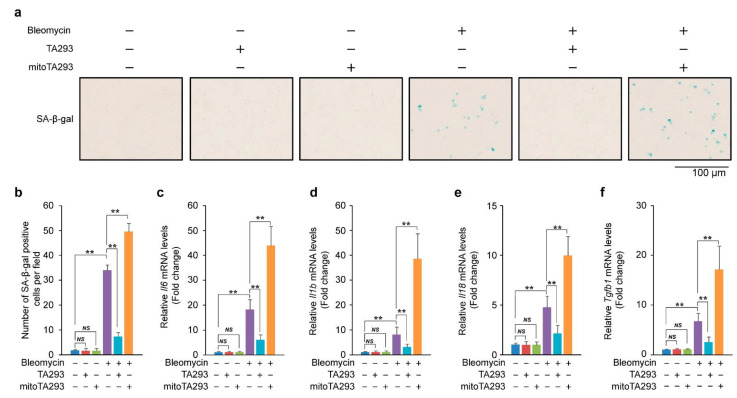
Effects of TA293 and mitoTA293 on cellular senescence in BLM-induced pulmonary fibrosis model mice. (**a**) Detection and (**b**) quantification of cellular senescence by SA-β-gal staining. Expression of the senescent cells secrete senescence-associated secretory phenotype (SASP) factors (**c**) *Il6*, (**d**) *Il1b*, (**e**) *Il18*, and (**f**) *Tgfb1* mRNA. The data are presented as the mean ± SEM (*n* = 5 per group); ** *p* < 0.01; *NS*, not significant.

Bleomycin induces cellular senescence [27]. Senescent cells secrete senescence-associated secretory phenotype (SASP) factors that induce and exacerbate inflammation and fibrosis [28,29,30]. Based on these findings, we examined the effects of TA293 and mitoTA293 on cellular senescence in the lungs of BLM-induced pulmonary fibrosis mice. In the lung, eight days after BLM administration, TA293 suppressed the increase in the number of SA-β-gal positive cells, but mitoTA293 further increased their number (Figure 2a). These results show that cyto ^•^OH induces cellular senescence, but mt ^•^OH suppresses the exacerbation of cellular senescence.

Next, we examined the expression of SASP factor mRNAs in the lungs of BLM-induced pulmonary fibrosis mice. In the lungs, eight days after BLM administration, TA293 suppressed the increase in the expression of SASP factor mRNAs Il6, Il1b, Il18, and Tgfb1, whereas mitoTA293 further increased their expression (Figure 2b–f).

Taken together, these results indicate that cyto ^•^OH causes cellular senescence and induces the secretion of SASP factors in the lungs of BLM-induced pulmonary fibrosis mice. In contrast, mt ^•^OH plays a role in suppressing the exacerbation of cellular senescence and reducing the secretion of SASP factors.

### 3.3. In BLM-Treated PAECs, TA293 Suppresses and mitoTA293 Exacerbates Cellular Senescence

Alveolar epithelial cells are composed of type I and type II alveolar epithelial cells and cover more than 99% of the surface area inside the lung [31]. In addition, in the lungs of the BLM-induced pulmonary fibrosis mouse model, cellular senescence is induced in alveolar epithelial cells [27]. In vitro, BLM induces cellular senescence, and senescent cells secrete SASP factors such as TGF-β and inflammatory cytokines [32,33].

**Figure 3:**
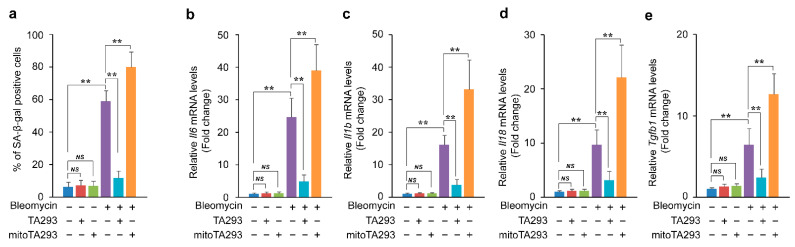
Effects of TA293 and mitoTA293 on oxidative stress and cellular senescence in BLM-induced primary pulmonary alveolar epithelial cells (PAECs) in vitro. (**a**) Quantification of cellular senescence in vitro. Expression of SASP factor mRNA: (**b**) *Il6*, (**c**) *Il1b*, (**d**) *Il18*, and (**e**) *Tgfb1*. PAECs were treated with saline or BLM and with vehicle, TA293, or mitoTA293 for 72 h. The data are presented as the mean ± SEM (*n* = 5 per group); ** *p* < 0.01; *NS*, not significant.

We examined the effects of TA293 and mitoTA293 on the induction of cellular senescence and the secretion of SASP factors in BLM-stimulated PAECs. In BLM-treated PAECs, TA293 suppressed the increase in the number of SA-βgal-positive cells, whereas mitoTA293 increased this number (Figure 3a). Furthermore, TA293 suppressed the upregulation of the SASP factors *Il6*, *Il1b*, *Il18*, and *Tgfb1* mRNAs, while mitoTA293 further upregulated their expression levels (Figure 3b–e).

Taken together, these results demonstrate that cyto ^•^OH causes cellular senescence and induces the secretion of SASP factors in BLM-stimulated PAECs. In contrast, mt ^•^OH suppresses the exacerbation of cellular senescence and secretion of SASP factors.

### 3.4. In BLM-Treated PAECs, TA293 Suppresses the Reduction of GSH in Cytoplasmic and Mitochondrial Fractions and Thereby, the Oxidative Damage to nDNA and mtDNA

**Figure 4:**
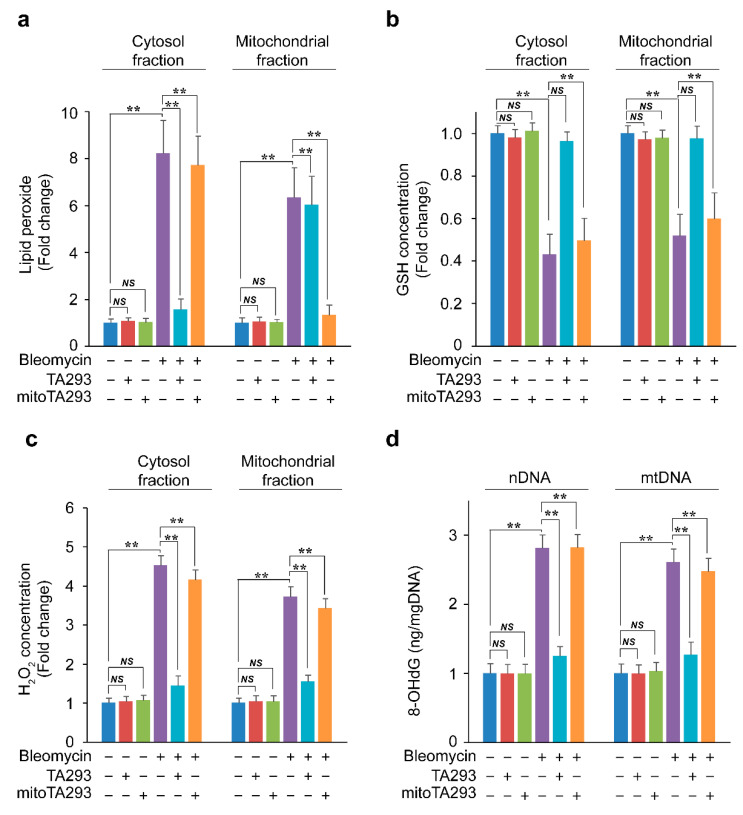
Effects of TA293 and mitoTA293 on the production of lipid peroxide, glutathione (GSH), H_2_O_2_, and oxidized DNA in cytosol and mitochondrial fractions in BLM-induced PAECs in vitro. Concentration of (**a**) lipid peroxide, (**b**) GSH, (**c**) H_2_O_2_, and (**d**) 8-OHdG in the cytosol and mitochondrial fractions in BLM-induced PAECs. PAECs were treated with saline or BLM and with vehicle, TA293, or mitoTA293 for 72 h. The data are presented as the mean ± SEM (*n* = 5 per group); ** *p* < 0.01; *NS*, not significant.

The ^•^OH ion is formed from hydrogen peroxide by a metal-catalyzed Fenton reaction at the water interface opposite a hydrophobic medium, such as the plasma membrane [34]. Therefore, it is speculated that cyto ^•^OH and mt ^•^OH are involved in the oxidation of lipid components in cell membranes and mitochondrial membranes, respectively. In BLM-treated PAECs, TA293 and mitoTA293 specifically suppressed the production of lipid peroxides in the cytoplasm and mitochondrial fractions, respectively (Figure 4a). These results indicate that cyto ^•^OH and mt ^•^OH produce lipid peroxides in the cytoplasm and mitochondrial fractions, respectively.

Hydrogen peroxide depletes intracellular GSH and increases H_2_O_2_ levels [35]. GSH is synthesized in the cytoplasm and translocates to the mitochondria via SLC25A11 [36]. In this study, TA293 suppressed the decrease in cytoplasmic fraction GSH (cytoGSH) and mitochondrial fraction GSH (mtGSH) in BLM-treated PAECs (Figure 4b). However, TA293 did not suppress the decrease in mtGSH in BLM-treated PAECs with knocked down SLC25A11 (Supplementary Figure S5a). mitoTA293 did not suppress the decrease in cytoGSH and mtGSH in BLM-treated PAECs but did suppress the decrease in mtGSH in antimycin A-treated PAECs (Figure 4b and Supplementary Figure S6b). These results indicate that cytoplasmic lipid peroxides produced by cyto ^•^OH reduce cytoGSH and mtGSH in BLM-treated PAECs. Furthermore, the results suggested that cytoGSH deficiency did not suppress the decrease in mtGSH, even if it suppressed the mitochondrial lipid peroxide produced by mt ^•^OH. These results indicate that cytoplasmic lipid peroxides produced by cyto ^•^OH reduce cytoGSH and mtGSH in BLM-treated PAECs. In BLM-treated PAECs, TA293 suppressed the enhancement of H_2_O_2_ in the cytoplasmic fraction (cytoH_2_O_2_) and the mitochondrial fraction (mtH_2_O_2_) but did not suppress the enhancement of mtH_2_O_2_ in *Slc25a11* knock-down BLM-treated PAECs. (Figure 4c and Supplementary Figure S5b). In contrast, mitoTA293 suppressed the enhancement of mtH_2_O_2_ in antimycin A-treated PAECs but did not suppress the enhancement of cytoH_2_O_2_ or mtH_2_O_2_ in BLM-treated PAECs (Figure 4c and Supplementary Figure S5c).

Hydrogen peroxide increases 8-OHdG in GSH-deficient cells [37]. In BLM-treated PAECs, TA293 suppressed the increase in 8-OHdG in nDNA and mtDNA (Figure 4d). TA293 suppressed the increase in 8-OHdG in nDNA and mtDNA in BLM-treated PAECs but did not suppress the increase in *Slc25a11* knock-down BLM-treated PAECs. In contrast, mitoTA293 suppressed the enhancement of 8-OHdG in mtDNA in antimycin A-treated PAECs but did not suppress such enhancement in nDNA or mtDNA in BLM-treated PAECs (Figure 4d and Supplementary Figure S5c).

Taken together, these findings suggest that cyto ^•^OH produces lipid peroxides in the cytoplasmic fraction and reduces cytoGSH and mtGSH in BLM-treated PAECs, which increases cytoH_2_O_2_ and mtH_2_O_2_ levels, resulting in oxidative damage to nDNA and mtDNA. However, mt ^•^OH produces lipid peroxide in the mitochondrial fraction but does not affect GSH reduction, H_2_O_2_ enhancement, or oxidative damage of mtDNA in the mitochondrial fraction.

### 3.5. In BLM-Treated PAECs, TA293 Suppresses and mitoTA293 Exacerbates Cellular Senescence by Different Pathways

**Figure 5:**
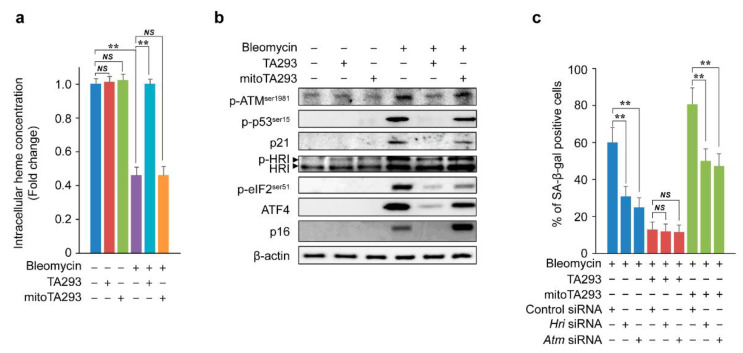
Effects of TA293 and mitoTA293 on p-ATM^ser1981^/p-p53^ser15^/p21 and p-HRI/p-eIF2/ATF4/p16 pathways in BLM-induced PAECs in vitro. (**a**) Intracellular heme concentration. (**b**) Expression of p21, ATF4, and p16 and phosphorylation of ATM^ser1981^, p53^ser15^, HRI, and eIF2^ser51^ visualized by western blotting. PAECs were treated with saline or BLM and with vehicle, TA293, or mitoTA293 for 72 h. (**c**) Effect of *Hri* and *Atm* siRNA on cellular senescence. The data are presented as the mean ± SEM (*n* = 5 per group); ** *p* < 0.01; *NS*, not significant.

Lipid peroxides easily react with heme proteins to produce reactive carbonyls, which directly reduce heme [38]. Heme proteins, such as GSH peroxidase and catalase, indirectly reduce heme by eliminating H_2_O_2_ increased by GSH deficiency [39]. Damage to DNA and heme reduction due to excess H_2_O_2_ activates the p-ATM^ser1981^/p-p53^ser15^/p21 and p-HRI/p-eIF2^ser51^/ATF4/p16 pathways to induce cellular senescence [40]. Based on these reports, we examined the effects of TA293 and mitoTA293 on the pathways that induce cellular senescence in BLM-treated PAECs. In these cells, TA293 suppressed the depletion of intracellular heme concentration, but mitoTA293 did not (Figure 5a). In BLM-stimulated PAECs, TA293 suppressed the activation of the p-ATM^ser1981^/p-p53^ser15^/p21 and p-HRI/p-eIF2^ser51^/ATF4/p16 pathways, but mitoTA293 did not (Figure 5b). Similarly, in the BLM-induced pulmonary fibrosis model mice, TA293 suppressed the activation of these pathways, but mitoTA293 did not (Supplementary Figure S7). In BLM-stimulated PAECs, *Hri* and *Atm* siRNAs reduced the number of SA-β-positive cells, but TA293 did not affect their number (Figure 5c). However, mitoTA293 reduced the number of these cells, but not to the same level as that by TA293 (Figure 5c).

Taken together, these results suggest that cyto ^•^OH indirectly causes oxidative damage to nDNA and depletion of heme and induces cellular senescence via the p-ATM^ser1981^/p-p53^ser15^/p21 and p-HRI/p-eIF2^ser51^/ATF4/p16 pathways. However, mt ^•^OH suppresses the exacerbation of cellular senescence via a different pathway.

### 3.6. In BLM-Treated PAECs, mitoTA293 Suppresses the Induction of Mitophagy and Exacerbates Cellular Senescence

**Figure 6:**
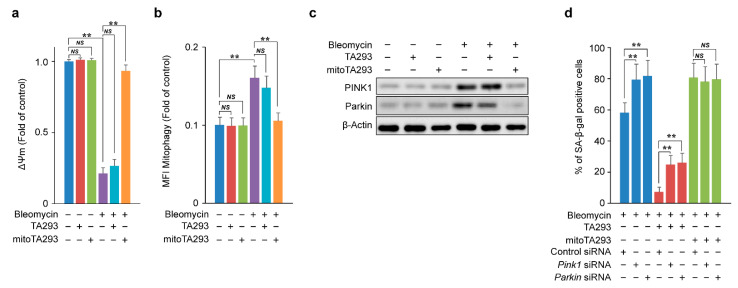
Effects of TA293 and mitoTA293 on mitochondria in BLM-induced PAECs in vitro. (**a**) Mitochondrial membrane potential (ΔΨm). (**b**) Quantification of mitophagy. (**c**) Expression of PINK1 and Parkin visualized by western blotting. (**d**) Effects of *Pink1* and *Parkin* siRNAs on cellular senescence. PAECs were treated with saline or BLM and with vehicle, TA293, or mitoTA293 for 72 h. The data are presented as the mean ± SEM (*n* = 5 per group); ** *p* < 0.01; *NS*, not significant.

In Figure 4a, the lipid peroxide in the mitochondrial fraction produced by mt ^•^OH is speculated to be derived from the mitochondrial membrane. Oxidative damage to the mitochondrial membrane causes depolarization of the mitochondrial membrane potential and induces mitophagy [41,42,43]. Based on these reports, we examined the effects of TA293 and mitoTA293 on mitochondrial membrane potential and mitophagy in BLM-treated PAECs. In these cells, TA293 did not suppress mitochondrial membrane depolarization, but mitoTA293 did (Figure 6a). In BLM-treated PAECs, TA293 did not suppress the induction of mitophagy, but mitoTA293 did (Figure 6b). In addition, TA293 did not suppress the expression of mitophagy inducers, such as PTEN-induced putative kinase 1 (PINK1) or E3 ubiquitin-protein ligase (Parkin), in BLM-stimulated PAECs, but mitoTA293 did (Figure 6c). Similarly, in the BLM-induced pulmonary fibrosis model mice, TA293 did not suppress PINK1 or Parkin expression, but mitoTA293 did (Supplementary Figure S8). Furthermore, *Pink1* and *Parkin* siRNA increased the number of SA-β-gal-positive cells in BLM-stimulated PAECs, but TA293 further increased their number (Figure 6d).

Taken together, these results suggest that, in BLM-stimulated PAECs, mt ^•^OH induces PINK1/Parkin-mediated mitophagy by depolarization due to peroxidation of the mitochondrial membrane and suppresses the exacerbation of cellular senescence, but cyto ^•^OH does not.

### 3.7. TA293 and mitoTA293 Have Opposite Effects on the NLRP3 Inflammasome/Caspase-1/IL1β/ILR/p-p38/p16 and p21 Pathways and Cellular Senescence

**Figure 7:**
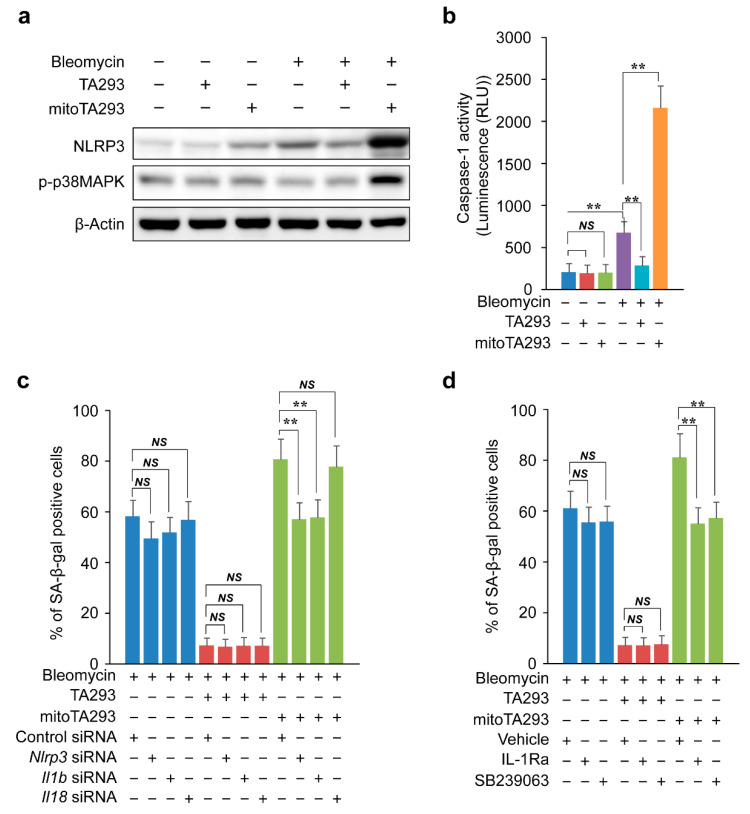
Effects of TA293 and mitoTA293 on NLRP3 inflammasome-induced cellular senescence in BLM-induced PAECs in vitro. (**a**) Expression of NLRP3 and phosphorylation of p38MAPK visualized by western blotting. (**b**) Caspase-1 activity based on luminescence analysis. (**c**) Effects of *Nlrp3*, *Il1b*, and *Il18* siRNAs on cellular senescence. (**d**) Effects of ILa1R and SB239063 on cellular senescence. PAPEs were treated with saline or BLM and with vehicle, TA293, or mitoTA293 for 72 h. The data are presented as the mean ± SEM (*n* = 5 per group); ** *p* < 0.01; *NS*, not significant.

Oxidized mtDNA induces activation of the NLRP3 inflammasome [44]. The NLRP3 inflammasome activates caspase-1 and induces the expression of IL-1β and IL-18 [45,46]. In BLM-stimulated PAECs, TA293 suppressed the expression of NLRP3 and activation of caspase-1, but mitoTA293 increased these effects (Figure 7a,b). Similarly, in the BLM-induced pulmonary fibrosis model mice, TA293 suppressed the expression of NLRP3, but mitoTA293 increased these effects (Supplementary Figure S8). In the BLM-induced pulmonary fibrosis model mice and BLM-stimulated PAECs, TA293 suppressed the expression of Il1b and Il18 mRNAs, but mitoTA293 increased their expression levels (Figure 2d,e and Figure 3c,d). *Nlrp3*, *Il1b*, and *Il18* siRNAs did not change the number of SA-β-gal-positive cells in BLM-treated PAECs (Figure 7c). Similarly, TA293 did not change the number of SA-β-gal-positive cells in siRNA-transfected BLM-treated PAECs (Figure 7c). mitoTA293 suppressed the increase in the number of SA-β-positive cells in *Nlrp3* and *Il1b* siRNA-transfected BLM-treated PAECs but did not suppress the increase in the number of such cells in *Il18* siRNA-transfected BLM-treated PAECs (Figure 7c).

Alveolar epithelial cells express IL1R [47]. IL-1β activates the MKK3/6 and p38MAPKα/β pathways [48]. In addition, p38MAPKα/β induces the expression of p16 and p21 [49]. In BLM-treated PAECs, TA293 did not affect the phosphorylation of p38 MAPK, but mitoTA293 increased it (Figure 7a). Similarly, in the BLM-induced pulmonary fibrosis model mice, TA293 did not affect the phosphorylation of p38 MAPK, but mitoTA293 increased it (Supplementary Figure S8). Furthermore, TA293 did not affect the number of SA-β-positive cells in BLM-treated PAECs with the IL1R inhibitor IL1Ra and the p38MAPKα/β inhibitor SB239063, but mitoTA293 suppressed the increase in their number (Figure 7d).

Taken together, these results indicate that oxidized mtDNA produced by cyto ^•^OH activates the NLRP3 inflammasome and induces the secretion of IL1-β and IL-18. IL1-β activates the IL-1R/p-p38 MAPK/p16 and p21 pathways in autocrine signaling, exacerbating cellular senescence. mt ^•^OH-induced mitophagy suppresses the activation of the NLRP3 inflammasome and exacerbation of cellular senescence via these pathways.

## 4. Discussion

Bleomycin produces ROS during DNA cleavage [50]. Since ROS plays an important role in the etiology of lung injury, it is considered one of the causes of lung fibrosis [6]. Among ROS, ^•^OH has been inferred to play a central role in lung injury and fibrosis formation because it has the strongest oxidizing power. Recent reports have shown that ^•^OH scavengers, such as hydrogen-rich saline and suplatast tosilate, suppress fibrosis formation [51,52]. However, in BLM-induced pulmonary fibrosis, the mechanism by which ^•^OH induces and exacerbates fibrosis has not been elucidated. In addition, the effect of differences in intracellular compartments, where ^•^OH is oxidatively damaged during fibrosis, is completely unknown. In this study, using TA293 and mitoTA293 in a BLM-induced pulmonary fibrosis model, we found that cyto ^•^OH and mt ^•^OH play different roles in fibrosis.

First, we clarified that cyto ^•^OH plays a role in inducing cellular senescence and inflammation and causing fibrosis in the lungs of BLM-induced pulmonary fibrosis model mice. Senescent cells secrete SASP factors, which induce inflammation and fibrosis, causing inflammation due to macrophage infiltration and tissue remodeling due to fibrosis [53]. Therefore, in BLM-induced pulmonary fibrosis model mice, senescent alveolar epithelial cells contribute to the development of pulmonary fibrosis; moreover, the removal of senescent alveolar epithelial cells by senolytic drugs suppresses the induction of fibrosis [54,55]. This study also suggested that cyto ^•^OH induces senescence in BLM-stimulated PAECs and induces inflammation and fibrosis by inducing the secretion of SASP factors. These findings are consistent with reports that NADPH oxidase and pyocyanin, which produce cyto ^•^OH, cause cellular senescence and induce inflammatory responses and fibrosis [23,56]. Excessive production of lipid peroxide significantly reduces intracellular GSH and enhances H_2_O_2_ production [57,58,59]. Excess H_2_O_2_ oxidatively damages DNA and induces cellular senescence via the p53/p21 pathway [60]. In this study, cyto ^•^OH produced lipid peroxides and reduced the amount of cyto GSH in alveolar epithelial cells. In addition, excess H_2_O_2_ caused by cyto GSH deficiency induced p21 expression via the ATM/p-p53^ser15^ pathway by oxidative damage to nDNA.

In contrast, lipid peroxides easily react with transition metal ions such as copper, iron, and heme proteins to produce active carbonyl compounds (reactive carbonyls) [38]. In this study, we consider that intracellular heme deficiency in BLM-treated PAECs causes cyto ^•^OH-generated lipid peroxides to directly reduce heme or heme proteins such as peroxidase and catalase, which reduce excess H_2_O_2_. Heme deficiency autophosphorylates HRI and induces the expression p16 via the p-eIF2^ser51^/ATF4 pathway [61,62,63]. Similarly, in this study, cyto ^•^OH-induced heme deficiency induced the expression of p16 via the p-HRI/p-eIF2^ser51^/ATF4 pathway. Furthermore, the mechanism by which cyto ^•^OH induces the expression of p16 and p21 to induce cellular senescence is consistent with the findings of studies using the ^•^OH scavenger hydrogen in the cyto OH-induced cellular senescence model [40].

Based on our findings, we concluded that mt ^•^OH-induced mitophagy suppresses the exacerbation of cellular senescence by eliminating the mitochondria in which cyto ^•^OH causes dysfunction. Oxidized mtDNA activates the NLRP3 inflammasome and promotes the secretion of IL-1β and IL-18 [44]. In this study, oxidized mtDNA produced by cyto ^•^OH increased the activation of NLRP3 inflammasome and IL-1β and IL-18 secretion. IL-1β activates the IL-1R/p38 MAPK/p16 and p21 pathways and induces cellular senescence [64]. In this study, in BLM-treated PAECs, IL-1β activated the IL-1R/p38 MAPK/p16 and p21 pathways, and IL-1ra and SB 239063 suppressed cellular senescence. These results are consistent with the findings that IL-1ra prevents or treats pulmonary fibrosis in BLM-induced mice, SB 239063 suppresses fibrosis, and p38 MAPKα induces cellular senescence [65,66,67,68]. On the contrary, our findings suggest that IL-18 secreted from PAECs is not involved in the induction of cellular senescence. Moreover, the results of this study suggest that IL-18 has no autocrine effect and is not involved in the induction of cellular senescence in PAECs. However, IL-18 accelerates fibroblast aging and exacerbates fibrosis in the lungs of mice with BLM-induced pulmonary fibrosis [69]. In addition, IL-18 causes macrophage activation syndrome and exacerbates inflammation [70,71]. Based on these findings, we suggest that IL-18 secreted from alveolar epithelial cells exacerbates fibrosis by inducing cell senescence of fibroblasts due to the paracrine effect and enhanced macrophage-mediated inflammation.

Unlike cyto ^•^OH, mt ^•^OH was suggested to attenuate cellular senescence and inflammation and suppress exacerbation of fibrosis in the lungs of BLM-induced pulmonary fibrosis model mice. In addition, mt ^•^OH produced the mitochondrial fraction lipid peroxides in BLM-treated alveolar epithelial cells. Depolarization of the mitochondrial membrane potential is caused by damage to the mitochondrial membrane and triggers the induction of mitophagy [72,73]. In this study, we considered that mt ^•^OH damaged and depolarized the mitochondrial membrane and induced mitophagy. Senescent cells show reduced mitophagy function [74]. In addition, mitophagy suppresses aging [75]. These findings suggest that mt ^•^OH-induced mitophagy eliminates mitochondria in which cyto ^•^OH causes dysfunction and oxidized mtDNA is increased. This is thought to suppress the activation of the NLRP inflammasome/caspase-1/IL1β/ILR/p-p38/p16 and p21 pathways and suppress the exacerbation of cellular senescence, inflammation, and fibrosis.

The limitation of this study is that the effects of cyto ^•^OH and mt ^•^OH on cells other than alveolar epithelial cells have not been clarified. Nevertheless, many studies have shown that cellular senescence of alveolar epithelial cells contributes to fibrosis. On the contrary, fibroblasts and macrophages other than alveolar epithelial cells are involved in the induction and exacerbation of fibrosis. Thus, clarifying the effects of cyto ^•^OH and mt ^•^OH on these cells may elucidate the mechanism of induction and exacerbation of fibrosis and contribute to better treatment and prevention. In particular, in this study, it is suggested that cyto ^•^OH is one of the inducers of pulmonary fibrosis. We believe that cyto ^•^OH scavengers such as TA293 have a protective effect on the onset and exacerbation of IPF.

In this study, we elucidated that ^•^OH localized in different intracellular compartments has opposite effects in inducing and suppressing pulmonary fibrosis. It is expected that the difference observed in this study with respect to the action of ^•^OH localized in intracellular compartments will contribute to the development of new preventive and therapeutic agents for pulmonary fibrosis.

## 5. Conclusions

This study involving BLM-induced pulmonary fibrosis mice and BLM-treated primary pulmonary alveolar epithelial cells suggests a model whereby the lipid peroxide produced by cyto ^•^OH causes a shortage of GSH and heme, increases H_2_O_2_, and induces cellular senescence via the ATM/p-p53^ser15^/p21 and p-HRI/p-elF2α/ATF4/p16 pathways. In addition, cytosol ^•^OH-induced depletion of cytosol GSH reduces mitochondrial GSH, produces H_2_O_2_ in the mitochondria and oxidized mitochondrial DNA, activates the NLRP inflammasome/caspase-1/IL1β/ILR/p-p38/p16 and p21 pathways, and exacerbates cellular senescence. In contrast, mitochondrial ^•^OH induces mitophagy and eliminates mitochondria that produce oxidized mitochondrial DNA, thereby suppressing the induction of the NLRP inflammasome and suppressing the exacerbation of cellular senescence. This is the first report of intracellularly localized ^•^OH having different functions.

## Data Availability

The data presented in this study are available in article and supplementary material.

## Supplementary Materials

The following are available online at https://www.mdpi.com/article/10.3390/antiox10091398/s1, Figure S1. Structures of TA293 and mitoTA293; Figure S2. Knockdown efficiency of each siRNA transfected into primary pulmonary alveolar epithelial cells (PAECs). Expression levels of siRNA target genes were quantified by qRT-PCR; Figure S3. Mice exposed to 20 mg/kg bleomycin (BLM) were sprayed with TA293 (10 mg/kg) or mitoTA293 (10 mg/kg) intratracheally once a day using a microsprayer from day 0 to day 7; Figure S4. Examination of the effects of TA293 and mitoTA293 on oxidative stress and inflammation in BLM-induced pulmonary fibrosis model mice based on in vivo imaging analysis. Representative images of (a) oxidative stress and (b) inflammation in the lungs of OKD48- and IDOL-Tg mice; Figure S5. Effects of TA293 and mitoTA293 on the production of lipid peroxide, GSH, H_2_O_2_, and oxidized DNA in the cytosol and mitochondrial fractions in BLM-induced PAECs in SLC25A11 siRNA-transfected mice. Concentrations of (a) GSH, (b) H_2_O_2_, and (c) 8-OHdG in the cytosol and mitochondrial fractions in BLM-induced PAECs. The data are presented as the mean ± SEM (*n* = 5 per group); ** *p* < 0.01; *NS*, not significant; Figure S6. Effects of TA293 and mitoTA293 on the production of lipid peroxide, GSH, H_2_O_2_, and oxidized DNA in the cytosol and mitochondrial fractions in antimycin A-induced PAECs. Concentrations of (a) lipid peroxide, (b) GSH, (c) H_2_O_2_, and (d) 8-OHdG in the cytosol and mitochondrial fractions in BLM-induced PAECs. The data are presented as the mean SEM (*n* = 5 per group); ** *p* < 0.01; *NS*, not significant; Figure S7. Effects of TA293 and mitoTA293 on p-ATM^ser1981^/p-p53^ser15^/p21 and p-HRI/p-eIF2/ATF4/p16 pathways in BLM-induced PAECs in the lungs of BLM-induced pulmonary fibrosis model mice. These expression levels were detected by western blotting analysis in the lungs of mice 8 days after BLM administration; Figure S8. Effects of TA293 and mitoTA293 on the expression of PINK1, Parkin, and the NLRP3 inflammasome and phosphorylation of p38MAPK in the lungs of BLM-induced pulmonary fibrosis model mice. These expression levels were detected by western blotting analysis in the lungs of mice 8 days after BLM administration.

## References

[1] Raghu G. (2017). Idiopathic pulmonary fibrosis: Lessons from clinical trials over the past 25 years. Eur. Respir. J..

[2] Buendía-Roldá I., Mejía M., Navarro C., Selman M. (2017). Idiopathic pulmonary fibrosis: Clinical behavior and aging associated comorbidities. Respir. Med..

[3] Liu Y.M., Nepali K., Liou J.P. (2017). Idiopathic pulmonary fibrosis: Current status, recent progress, and emerging targets. J. Med. Chem..

[4] Raghu G., Weycker D., Edelsberg J., Bradford W.Z., Oster G. (2006). Incidence and prevalence of idiopathic pulmonary fibrosis. Am. J. Respir. Crit. Care Med..

[5] Pérez E.R.F., Daniels C.E., Schroeder D.R., St Sauver J., Hartman T.E., Bartholmai B.J., Yi E.S., Ryu J.H. (2010). Incidence, prevalence, and clinical course of idiopathic pulmonary fibrosis: A population-based study. Chest.

[6] Cheresh P., Kim S.J., Tulasiram S., Kamp D.W. (2013). Oxidative stress and pulmonary fibrosis. Biochim. Biophys. Acta.

[7] Li Y.J., Shimizu T., Shinkai Y., Hirata Y., Inagaki H., Takeda K., Azuma A., Yamamoto M., Kawada T. (2017). Nrf2 regulates the risk of a diesel exhaust inhalation-induced immune response during bleomycin lung injury and fibrosis in mice. Int. J. Mol. Sci..

[8] Moeller A., Ask K., Warburton D., Gauldie J., Kolb M. (2008). The bleomycin animal model: A useful tool to investigate treatment options for idiopathic pulmonary fibrosis?. Int. J. Biochem. Cell Biol..

[9] Highfield J.A., Mehta L.K., Parrick J., Wardman P. (2000). Synthesis, hydroxyl radical production and cytotoxicity of analogues of bleomycin. Bioorg. Med. Chem..

[10] Allawzi A., Elajaili H., Redente E.F., Nozik-Grayck E. (2019). Oxidative toxicology of bleomycin: Role of the extracellular redox environment. Curr. Opin. Toxicol..

[11] Suryadevara V., Ramchandran R., Kamp D.W., Natarajan V. (2020). Lipid mediators regulate pulmonary fibrosis: Potential mechanisms and signaling pathways. Int. J. Mol. Sci..

[12] Li X., Zhang W., Cao Q., Wang Z., Zhao M., Xu L., Zhuang Q. (2020). Mitochondrial dysfunction in fibrotic diseases. Cell Death Discov..

[13] Bigarella C.L., Liang R., Ghaffari S. (2014). Stem cells and the impact of ROS signaling. Development.

[14] Bowler R.P., Nicks M., Warnick K., Crapo J.D. (2002). Role of extracellular superoxide dismutase in bleomycin-induced pulmonary fibrosis. Am. J. Physiol. Lung Cell. Mol. Physiol..

[15] Cortijo J., Cerdá-Nicolás M., Serrano A., Bioque G., Estrela J.M., Santangelo F., Esteras A., Llombart-Bosch A., Morcillo E.J. (2001). Attenuation by oral N-acetylcysteine of bleomycin-induced lung injury in rats. Eur. Respir. J..

[16] Anathy V., Lahue K.G., Chapman D.G., Chia S.B., Casey D.T., Aboushousha R., van der Velden J.L.J., Elko E., Hoffman S.M., McMillan D.H. (2018). Reducing protein oxidation reverses lung fibrosis. Nat. Med..

[17] Sakai T., Imai J., Ito T., Takagaki H., Ui M., Hatta S. (2017). The novel antioxidant TA293 reveals the role of cytoplasmic hydroxyl radicals in oxidative stress-induced senescence and inflammation. Biochem. Biophys. Res. Commun..

[18] Ohsawa I., Ishikawa M., Takahashi M., Watanabe M., Nishimaki K., Yamagata K., Katsura K.I., Katayama Y., Asoh S., Ohta S. (2007). Hydrogen acts as a therapeutic antioxidant by selectively reducing cytotoxic oxygen radicals. Nat. Med..

[19] Kayar S.R., Axley M.J., Homer L.D., Harabin A.L. (1994). Hydrogen gas is not oxidized by mammalian tissues under hyperbaric conditions. Undersea Hyperb. Med..

[20] Turnbull S., Tabner B.J., El-Agnaf O.M., Moore S., Davies Y., Allsop D. (2001). alpha-Synuclein implicated in Parkinson’s disease catalyses the formation of hydrogen peroxide in vitro. Free Radic. Biol. Med..

[21] Marlatt M., Lee H.G., Perry G., Smith M.A., Zhu X. (2004). Sources and mechanisms of cytoplasmic oxidative damage in Alzheimer’s disease. Acta Neurobiol. Exp..

[22] Schaar C.E., Dues D.J., Spielbauer K.K., Machiela E., Cooper J.F., Senchuk M., Hekimi S., van Raamsdonk J.M. (2015). Mitochondrial and cytoplasmic ROS have opposing effects on lifespan. PLoS Genet..

[23] Sakai T., Imai J., Takagaki H., Ui M., Hatta S. (2019). Cytoplasmic OH scavenger TA293 attenuates cellular senescence and fibrosis by activating macrophages through oxidized phospholipids/TLR4. Life Sci..

[24] Oikawa D., Akai R., Tokuda M., Iwawaki T. (2012). A transgenic mouse model for monitoring oxidative stress. Sci. Rep..

[25] Iwawaki T., Akai R., Oikawa D., Toyoshima T., Yoshino M., Suzuki M., Takeda N., Ishikawa T., Kataoka Y., Yamamura K.I. (2015). Transgenic mouse model for imaging of interleukin-1β-related inflammation in vivo. Sci. Rep..

[26] Chaudhary N.I., Schnapp A., Park J.E. (2006). Pharmacologic differentiation of inflammation and fibrosis in the rat bleomycin model. Am. J. Respir. Crit. Care Med..

[27] Aoshiba K., Tsuji T., Nagai A. (2003). Bleomycin induces cellular senescence in alveolar epithelial cells. Eur. Respir. J..

[28] Coppé J.P., Patil C.K., Rodier F., Sun Y., Muñoz D.P., Goldstein J., Nelson P.S., Desprez P.Y., Campisi J. (2008). Senescence-associated secretory phenotypes reveal cell-nonautonomous functions of oncogenic RAS and the p53 tumor suppressor. PLoS Biol..

[29] Anåker A., von Koch L., Sjöstrand C., Bernhardt J., Elf M.A. (2017). comparative study of patients’ activities and interactions in a stroke unit before and after reconstruction-The significance of the built environment. PLoS ONE.

[30] Jun J.I., Lau L.F. (2010). Cellular senescence controls fibrosis in wound healing. Aging.

[31] Crapo J.D., Young S.L., Fram E.K., Pinkerton K.E., Barry B.E., Crapo R.O. (1983). Morphometric characteristics of cells in the alveolar region of mammalian lungs. Am. Rev. Respir. Dis..

[32] Chen K.J., Li Q., Wen C.M., Duan Z.X., Zhang J.Y., Xu C., Wang J.M. (2016). Bleomycin (BLM) induces epithelial-to-mesenchymal transition in cultured A549 cells via the TGF-β/Smad signaling pathway. J. Cancer.

[33] Sun L., Chiang J.Y., Choi J.Y., Xiong Z.M., Mao X., Collins F.S., Hodes R.J., Cao K. (2019). Transient induction of telomerase expression mediates senescence and reduces tumorigenesis in primary fibroblasts. Proc. Natl. Acad. Sci. USA.

[34] Enami S., Sakamoto Y., Colussi A.J. (2014). Fenton chemistry at aqueous interfaces. Proc. Natl. Acad. Sci. USA.

[35] Rhee S.G., Cho C.S. (2010). Blot-based detection of dehydroalanine-containing glutathione peroxidase with the use of biotin-conjugated cysteamine. Methods Enzymol..

[36] Lushchak V.I. (2012). Glutathione homeostasis and functions: Potential targets for medical interventions. J. Amino Acids.

[37] Abu-Shakra A., Zeiger E. (1997). Formation of 8-hydroxy-2′-deoxyguanosine following treatment of 2′-deoxyguanosine or DNA by hydrogen peroxide or glutathione. Mutat. Res..

[38] Han A.P., Yu C., Lu L., Fujiwara Y., Browne C., Chin G., Fleming M., Leboulch P., Orkin S.H., Chen J.J. (2001). Heme-regulated eIF2alpha kinase (HRI) is required for translational regulation and survival of erythroid precursors in iron deficiency. EMBO J..

[39] Bowman S.E.J., Bren K.L. (2008). The chemistry and biochemistry of heme c: Functional bases for covalent attachment. Nat. Prod. Rep..

[40] Sakai T., Kurokawa R., Hirano S.I., Imai J. (2019). Hydrogen indirectly suppresses increases in hydrogen peroxide in cytoplasmic hydroxyl radical-induced cells and suppresses cellular senescence. Int. J. Mol. Sci..

[41] Bertin G., Averbeck D. (2006). Cadmium: Cellular effects, modifications of biomolecules, modulation of DNA repair and genotoxic consequences (a review). Biochimie.

[42] Shiba-Fukushima K., Imai Y., Yoshida S., Ishihama Y., Kanao T., Sato S., Hattori N. (2012). PINK1-mediated phosphorylation of the Parkin ubiquitin-like domain primes mitochondrial translocation of Parkin and regulates mitophagy. Sci. Rep..

[43] Matsuda N., Sato S., Shiba K., Okatsu K., Saisho K., Gautier C.A., Sou Y.S., Saiki S., Kawajiri S., Sato F. (2010). PINK1 stabilized by mitochondrial depolarization recruits Parkin to damaged mitochondria and activates latent Parkin for mitophagy. J. Cell Biol..

[44] Shimada K., Crother T.R., Karlin J., Dagvadorj J., Chiba N., Chen S., Ramanujan V.K., Wolf A.J., Vergnes L., Ojcius D.M. (2012). Oxidized mitochondrial DNA activates the NLRP3 inflammasome during apoptosis. Immunity.

[45] Núñez G. (2011). Intracellular sensors of microbes and danger. Immunol. Rev..

[46] Strowig T., Henao-Mejia J., Elinav E., Flavell R. (2012). Inflammasomes in health and disease. Nature.

[47] Bello-Irizarry S.N., Wang J., Olsen K., Gigliotti F., Wright T.W. (2012). The alveolar epithelial cell chemokine response to pneumocystis requires adaptor molecule MyD88 and interleukin-1 receptor but not toll-like receptor 2 or 4. Infect. Immun..

[48] Sakai A., Han J., Cato A.C.B., Akira S., Li J.D. (2004). Glucocorticoids synergize with IL-1beta to induce TLR2 expression via MAP Kinase Phosphatase-1-dependent dual Inhibition of MAPK JNK and p38 in epithelial cells. BMC Mol. Biol..

[49] Xu Y., Li N., Xiang R., Sun P. (2014). Emerging roles of the p38 MAPK and PI3K/AKT/mTOR pathways in oncogene-induced senescence. Trends Biochem. Sci..

[50] Burger R.M., Peisach J., Blumberg W.E., Horwitz S.B. (1979). Iron-bleomycin interactions with oxygen and oxygen analogues. Effects on spectra and drug activity. J. Biol. Chem..

[51] Dong W.W., Zhang Y.Q., Zhu X.Y., Mao Y.F., Sun X.J., Liu Y.J., Jiang L. (2017). Protective effects of hydrogen-rich saline against lipopolysaccharide-induced alveolar epithelial-to-mesenchymal transition and pulmonary fibrosis. Med. Sci. Monit..

[52] Fukuhara K., Nakashima T., Abe M., Masuda T., Hamada H., Iwamoto H., Fujitaka K., Kohno N., Hattori N. (2017). Suplatast tosilate protects the lung against hyperoxic lung injury by scavenging hydroxyl radicals. Free Radic. Biol. Med..

[53] Schafer M.J., Haak A.J., Tschumperlin D.J., LeBrasseur N.K. (2018). Targeting senescent cells in fibrosis: Pathology, paradox, and practical considerations. Curr. Rheumatol. Rep..

[54] Schafer M.J., White T.A., Iijima K., Haak A.J., Ligresti G., Atkinson E.J., Oberg A.L., Birch J., Salmonowicz H., Zhu Y. (2017). Cellular senescence mediates fibrotic pulmonary disease. Nat. Commun..

[55] Lehmann M., Korfei M., Mutze K., Klee S., Skronska-Wasek W., Alsafadi H.N., Ota C., Costa R., Schiller H.B., Lindner M. (2017). Senolytic drugs target alveolar epithelial cell function and attenuate experimental lung fibrosis ex vivo. Eur. Respir. J..

[56] Hecker L., Vittal R., Jones T., Jagirdar R., Luckhardt T.R., Horowitz J.C., Pennathur S., Martinez F.J., Thannickal V.J. (2009). NADPH oxidase-4 mediates myofibroblast activation and fibrogenic responses to lung injury. Nat. Med..

[57] Lei X.G., Cheng W.H., McClung J.P. (2007). Metabolic regulation and function of glutathione peroxidase-1. Annu. Rev. Nutr..

[58] Brigelius-Flohé R., Maiorino M. (2013). Glutathione peroxidases. Biochim. Biophys. Acta.

[59] Smeyne M., Smeyne R.J. (2013). Glutathione metabolism and Parkinson’s disease. Free Radic. Biol. Med..

[60] Shi T., Dansen T.B. (2020). Reactive oxygen species induced p53 activation: DNA damage, redox signaling, or both?. Antioxid. Redox Signal..

[61] Semchyshyn H.M. (2014). Reactive carbonyl species in vivo: Generation and dual biological effects. Sci. World J..

[62] Liu J., Yang J.R., Chen X.M., Cai G.Y., Lin L.R., He Y.N. (2015). Impact of ER stress-regulated ATF4/p16 signaling on the premature senescence of renal tubular epithelial cells in diabetic nephropathy. Am. J. Physiol. Cell. Physiol..

[63] Donnelly N., Gorman A.M., Gupta S., Samali A. (2013). The eIF2α kinases: Their structures and functions. Cell. Mol. Life Sci..

[64] Thornton T.M., Rincon M. (2009). Non-classical p38 map kinase functions: Cell cycle checkpoints and survival. Int. J. Biol. Sci..

[65] Piguet P.F., Vesin C., Grau G.E., Thompson R.C. (1993). Interleukin 1 receptor antagonist (IL-1ra) prevents or cures pulmonary fibrosis elicited in mice by bleomycin or silica. Cytokine.

[66] Borthwick L.A. (2016). The IL-1 cytokine family and its role in inflammation and fibrosis in the lung. Semin. Immunopathol..

[67] Underwood D.C., Osborn R.R., Bochnowicz S., Webb E.F., Rieman D.J., Lee J.C., Romanic A.M., Adams J.L., Hay D.W., Griswold D.E. (2000). SB 239063, a p38 MAPK inhibitor, reduces neutrophilia, inflammatory cytokines, MMP-9, and fibrosis in lung. Am. J. Physiol. Lung Cell. Mol. Physiol..

[68] Luo Y., Zou P., Zou J., Wang J., Zhou D., Liu L. (2011). Autophagy regulates ROS-induced cellular senescence via p21 in a p38 MAPKα dependent manner. Exp. Gerontol..

[69] Zhang L.M., Zhang J., Zhang Y., Fei C., Wang L., Yi Z.W., Zhang Z.Q. (2019). Interleukin-18 promotes fibroblast senescence in pulmonary fibrosis through down-regulating Klotho expression. Biomed. Pharmacother..

[70] Girard-Guyonvarc C., Palomo J., Martin P., Rodriguez E., Troccaz S., Palmer G., Gabay C. (2018). Unopposed IL-18 signaling leads to severe TLR9-induced macrophage activation syndrome in mice. Blood.

[71] Weiss E.S., Girard-Guyonvarc’h C., Holzinger D., de Jesus A.A., Tariq Z., Picarsic J., Schiffrin E.J., Foell D., Grom A.A., Ammann S. (2018). Interleukin-18 diagnostically distinguishes and pathogenically promotes human and murine macrophage activation syndrome. Blood.

[72] Ashrafi G., Schwarz T.L. (2013). The pathways of mitophagy for quality control and clearance of mitochondria. Cell Death Differ..

[73] Ding W.X., Yin X.M. (2012). Mitophagy: Mechanisms, pathophysiological roles, and analysis. Biol. Chem..

[74] Korolchuk V.I., Miwa S., Carroll B., von Zglinicki T. (2017). Mitochondria in cell senescence: Is mitophagy the weakest link?. EBioMedicine.

[75] Araya J., Tsubouchi K., Sato N., Ito S., Minagawa S., Hara H., Hosaka Y., Ichikawa A., Saito N., Kadota T. (2019). PRKN-regulated mitophagy and cellular senescence during COPD pathogenesis. Autophagy.

