# Supplementary material for: Effects of the Cytoplasm and Mitochondrial Specific Hydroxyl Radical Scavengers TA293 and mitoTA293 in Bleomycin-Induced Pulmonary Fibrosis Model Mice
Source: Antioxidants (Basel). 2021 Aug 31;10(9):1398. doi: 10.3390/antiox10091398 (PMC8469049; doi:10.3390/antiox10091398)
Supplement: Supplementary file 1 [file antioxidants-10-01398-s001.zip › antioxidants-1264481-supplementary.pdf]

Supplementary Materials

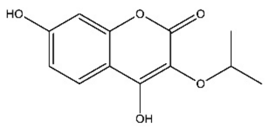

TA293

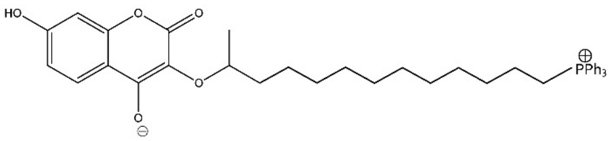

mito TA293

Figure S1. Structures of TA293 and mitoTA293.

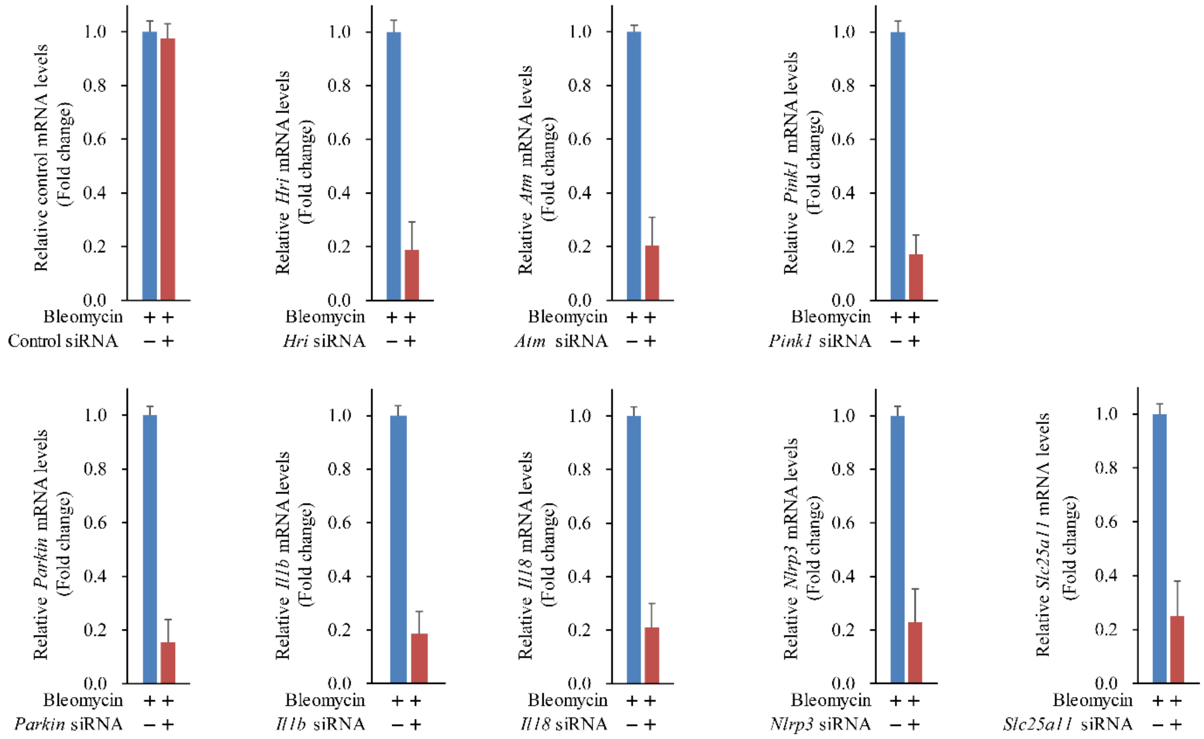

Figure S2. Knockdown efficiency of each siRNA transfected into primary pulmonary alveolar epithelial cells (PAECs). Expression levels of siRNA target genes were quantified by qRT-PCR.

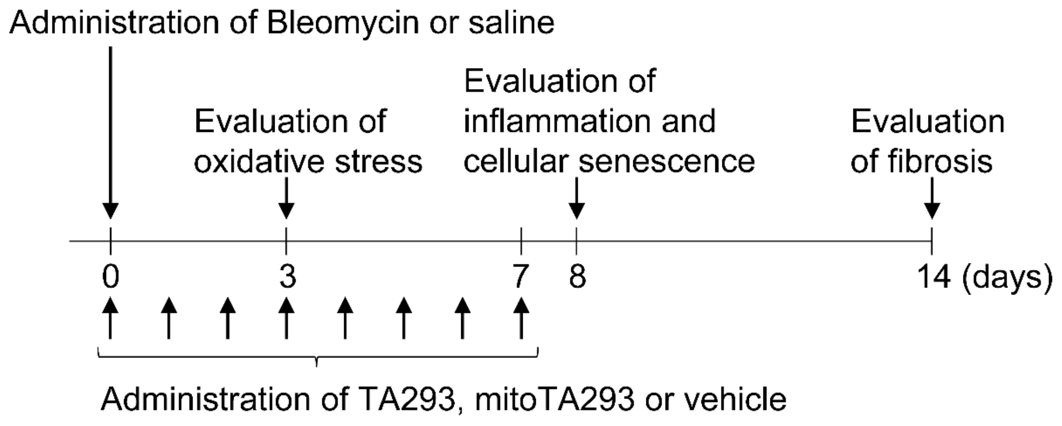

Figure S3. Mice exposed to 20 mg/kg bleomycin (BLM) were sprayed with TA293 (10 mg/kg) or mitoTA293 (10 mg/kg) intratracheally once a day using a microsprayer from day 0 to day 7.

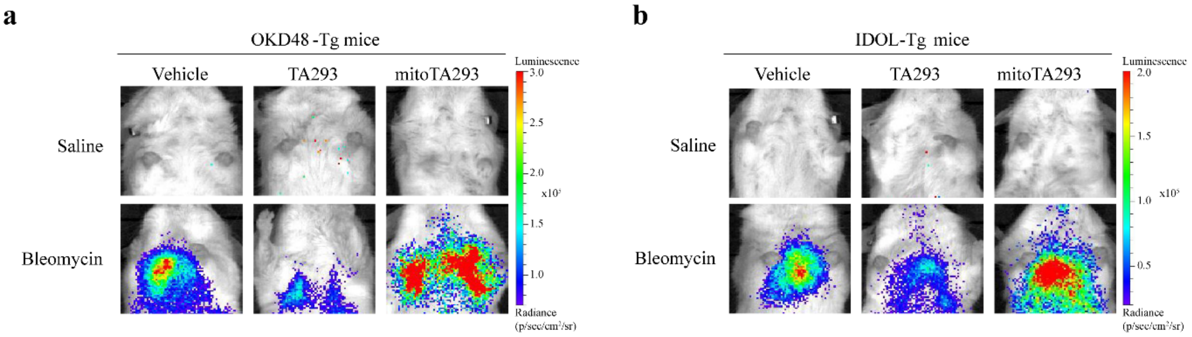

Figure S4. Examination of the effects of TA293 and mitoTA293 on oxidative stress and inflammation in BLM-induced pulmonary fibrosis model mice based on *in vivo* imaging. Representative images of (a) oxidative stress and (b) inflammation in the lungs of OKD48- and IDOL-Tg mice.

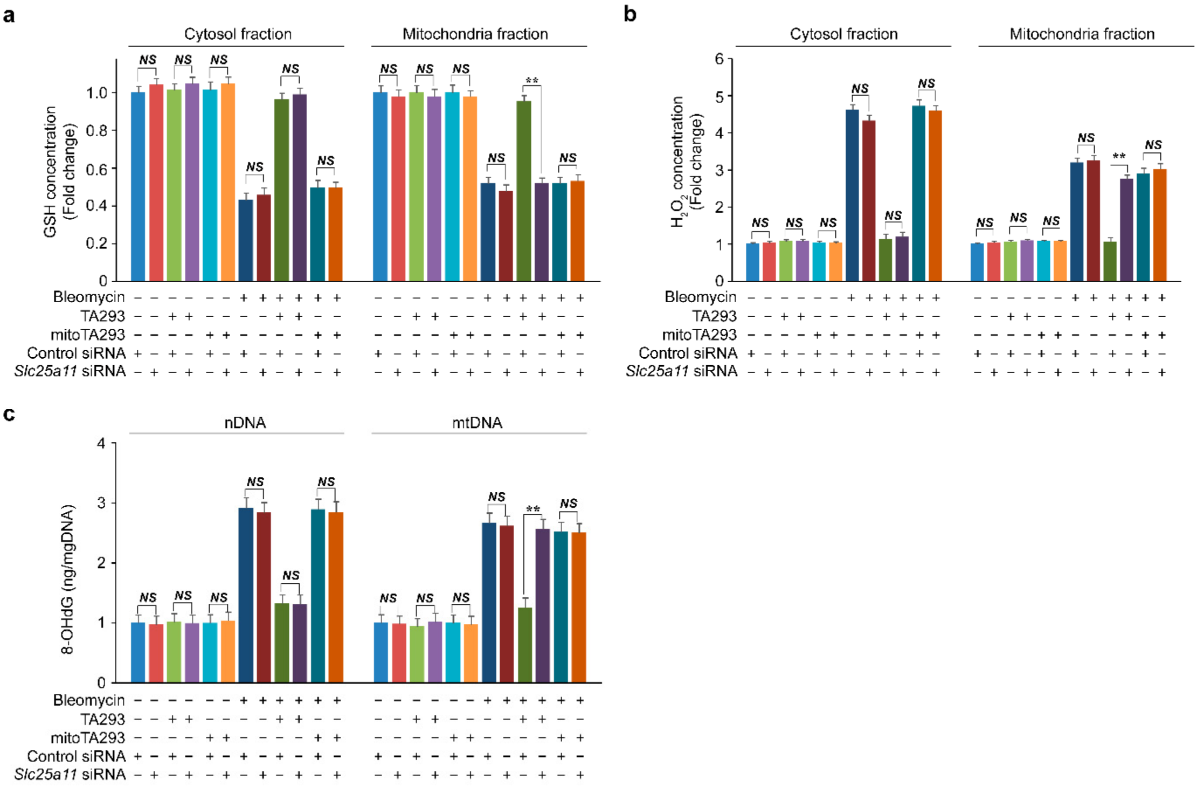

Figure S5. Effects of TA293 and mitoTA293 on the production of lipid peroxide, glutathione (GSH), H_2O_2, and oxidized DNA in the cytosol and mitochondrial fractions in BLM-induced PAECs in SLC25A11 siRNA-transfected mice. Concentrations of (a) GSH, (b) H_2O_2, and (c) 8-OHdG in the cytosol and mitochondrial fractions in BLM-induced PAECs. The data are presented as the mean SEM ($n = 5$ per group); ** $p < 0.01$; *NS*, not significant.

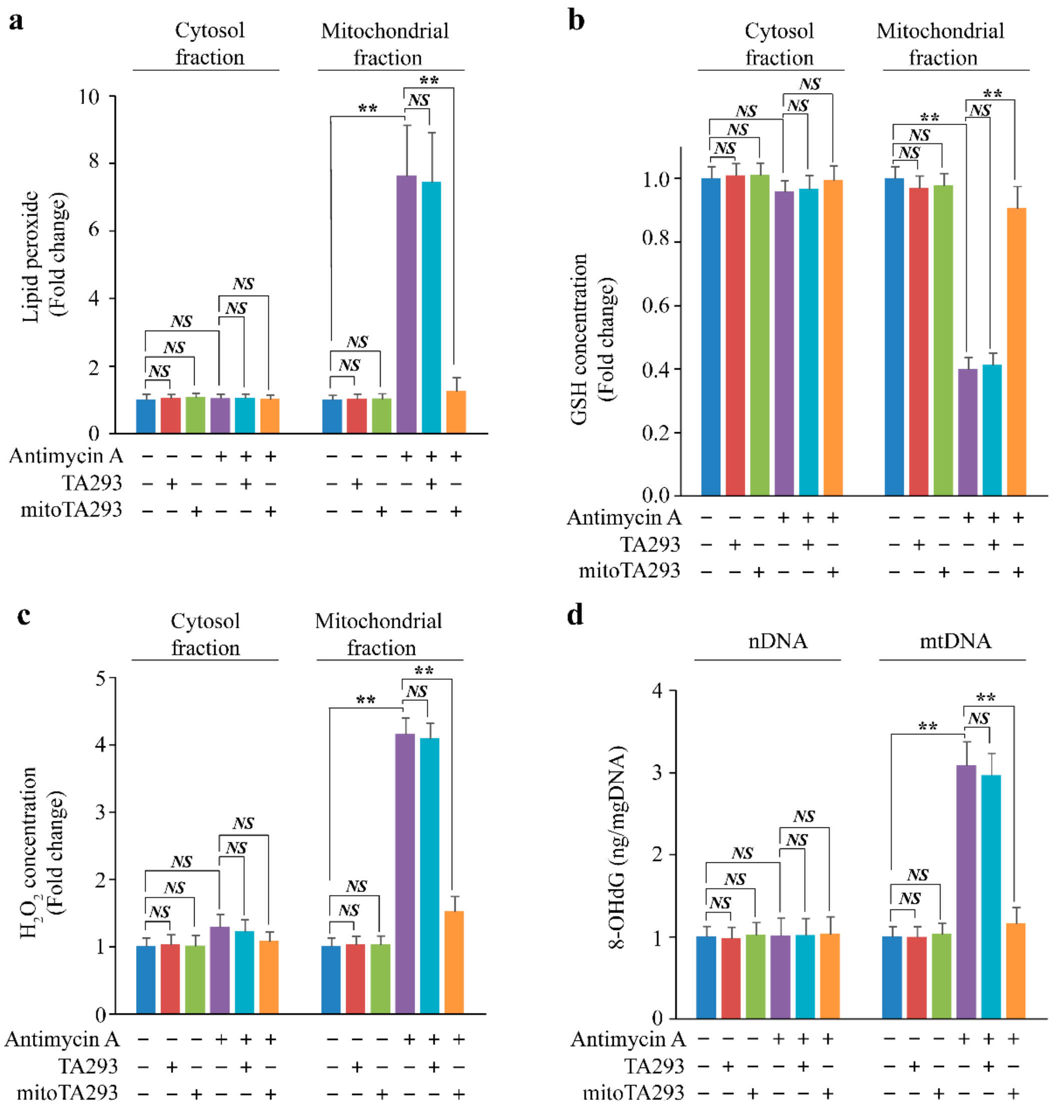

Figure S6. Effects of TA293 and mitoTA293 on the production of lipid peroxide, GSH, H_2O_2, and oxidized DNA in the cytosol and mitochondrial fractions in antimycin A-induced PAECs. Concentrations of (a) lipid peroxide, (b) GSH, (c) H_2O_2, and (d) 8-OHdG in the cytosol and mitochondrial fractions in BLM-induced PAECs. The data are presented as the mean SEM (n = 5 per group); ** $p < 0.01$; *NS*, not significant.

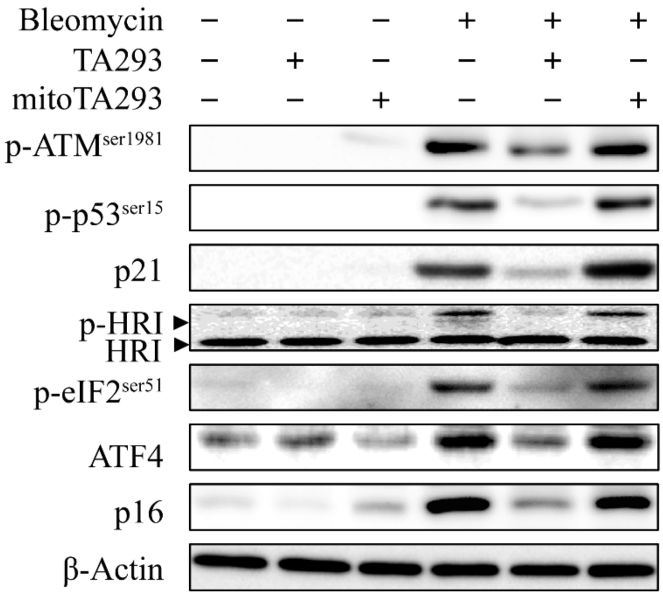

Figure S7. Effects of TA293 and mitoTA293 on p-ATM^{ser1981}/p-p53^{ser15}/p21 and p-HRI/p-eIF2^{ser51}/ATF4/p16 pathways in BLM-induced PAECs in the lungs of BLM-induced lung fibrosis model mice. These expression levels were detected by western blotting analysis in the lungs of mice 8 days after BLM administration.

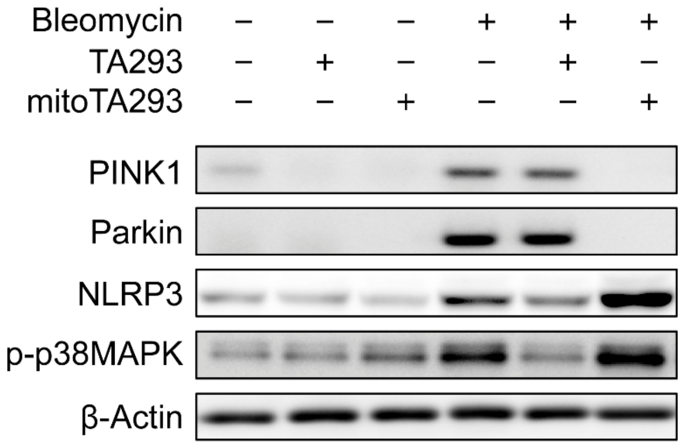

Figure S8. Effects of TA293 and mitoTA293 on the expression of PINK1, Parkin, and the NLRP3 inflammasome and phosphorylation of p38MAPK in the lungs of BLM-induced lung fibrosis model mice. These expression levels were detected by western blotting analysis in the lungs of mice 8 days after BLM administration.
